# Monophasic versus biphasic defibrillation for pediatric out-of-hospital cardiac arrest patients: a nationwide population-based study in Japan

**DOI:** 10.1186/cc11864

**Published:** 2012-11-13

**Authors:** Seizan Tanabe, Hideo Yasunaga, Soichi Koike, Manabu Akahane, Toshio Ogawa, Hiromasa Horiguchi, Tetsuo Hatanaka, Hiroyuki Yokota, Tomoaki Imamura

**Affiliations:** 1 Department of Emergency & Critical Care Medicine, Nippon Medical School 1-1-5 Sendagi, Bunkyo-ku Tokyo 113-8603, Japan; 2 Department of Health Management and Policy, Graduate School of Medicine, The University of Tokyo, 7-3-1 Hongo, Bunkyo-ku, Tokyo 113-8655, Japan; 3 Department of Planning, Information and Management, The University of Tokyo Hospital, 7-3-1 Hongo, Bunkyo-ku, Tokyo 113-8655, Japan; 4 Department of Public Health, Health Management and Policy, Nara Medical University School of Medicine, 840 Shijocho, Kashihara, Nara 634-8521, Japan; 5 Foundation for Ambulance Service Development, Emergency Life-Saving Technique Academy of Kyushu, 3-8-1 Ohura, Yahatanishi-ku, Kitakyushu, Fukuoka 807-0874, Japan

## Abstract

**Introduction:**

Conventional monophasic defibrillators for out-of-hospital cardiac-arrest patients have been replaced with biphasic defibrillators. However, the advantage of biphasic over monophasic defibrillation for pediatric out-of-hospital cardiac-arrest patients remains unknown. This study aimed to compare the survival outcomes of pediatric out-of-hospital cardiac-arrest patients who underwent monophasic defibrillation with those who underwent biphasic defibrillation.

**Methods:**

This prospective, nationwide, population-based observational study included pediatric out-of-hospital cardiac-arrest patients from January 1, 2005, to December 31, 2009. The primary outcome measure was survival at 1 month with minimal neurologic impairment. The secondary outcome measures were survival at 1 month and the return of spontaneous circulation before hospital arrival. Multivariable logistic regression analysis was performed to identify the independent association between defibrillator type (monophasic or biphasic) and outcomes.

**Results:**

Among 5,628 pediatric out-of-hospital cardiac-arrest patients (1 through 17 years old), 430 who received defibrillation shock with monophasic or biphasic defibrillator were analyzed. The number of patients who received defibrillation shock with monophasic defibrillator was 127 (30%), and 303 (70%) received defibrillation shock with biphasic defibrillator. The survival rates at 1 month with minimal neurologic impairment were 17.5% and 24.4%, the survival rates at 1 month were 32.3% and 35.6%, and the rates of return of spontaneous circulation before hospital arrival were 24.4% and 27.4% in the monophasic and biphasic defibrillator groups, respectively. Hierarchic logistic regression analyses by using generalized estimation equations found no significant difference between the two groups in terms of 1-month survival with minimal neurologic impairment (odds ratio (OR), 1.57; 95% confidence interval (CI), 0.87 to 2.83; *P *= 0.14) and 1-month survival (OR, 1.38; 95% CI, 0.87 to 2.18; *P *= 0.17).

**Conclusions:**

The present nationwide population-based observational study could not confirm an advantage of biphasic over monophasic defibrillators for pediatric OHCA patients.

## Introduction

Ventricular fibrillation (VF) accounts for 5% to 15% of out-of-hospital cardiac arrests (OHCAs) in children [1-4]. In such patients, early defibrillation may improve the outcome [4,5]. Depending on the wave pattern used in the defibrillation shock for VF, two types of defibrillator are used: monophasic and biphasic. It was reported that biphasic waveform defibrillation was more effective and safer compared with monophasic waveform defibrillation in controlled laboratory [6,7], in-hospital [8,9] and out-of-hospital settings [10-12]. In addition, biphasic defibrillators are smaller and lighter than monophasic defibrillators [13]. For these reasons, conventional monophasic defibrillators have been gradually replaced with biphasic defibrillators worldwide.

However, human studies comparing the two types of defibrillators have been performed only with adult patients. Even in a controlled laboratory setting or in-hospital setting, to our knowledge, no data compare the outcomes of defibrillation with different waveforms for pediatric OHCA patients. Thus, the advantage of a biphasic over a monophasic defibrillator for pediatric OHCA patients remains unknown.

In the present study, we used the nationwide OHCA registry database in Japan [14] to compare neurologically intact survival outcomes of pediatric OHCA patients who underwent defibrillation with a monophasic or biphasic defibrillator.

## Materials and methods

### Study design and data source

Our study used the All-Japan Utstein Registry database, which is a prospective, nationwide, population-based registry system of OHCA patients who are transferred to hospital by emergency medical service (EMS) providers [14]. Because only one nationwide emergency transport service system exists in Japan, all OHCA cases in which the patients were transported to hospital are registered in this database. Except for those with decapitation, incineration, decomposition, rigor mortis, or dependent cyanosis, almost all OHCA patients who are treated by EMS personnel are transported to hospital [14]. Therefore, patients in this database are representative of all OHCA patients in Japan.

The present study enrolled all pediatric patients aged 1 to 17 years who were transported to hospital by EMS personnel during 5 consecutive years from January 1, 2005, through December 31, 2009. In this period, because EMS personnel are allowed to perform defibrillation only on patients aged 1 year or older, the subjects in this study were those aged 1 year or older. Given the anonymous nature of the data, informed consent was waived. The study was approved by the Ethics Committee of Nara Medical University.

### EMS system in Japan

Japan has approximately 128 million residents (as of 2007) in an area of approximately 378,000 km^2^, and residents aged 17 years or younger account for 16% (21 million) of the population. The universal emergency telephone number of 119 directly connects to the regional fire defense headquarters. On acceptance of an emergency call, the nearest available ambulance is dispatched to a scene. All expenses are covered by the local government; the patient does not bear emergency treatment or transportation expenses. Except for limited areas where physician-manned ambulances or helicopters are available, the EMS system is a one-tiered response system [15].

Each ambulance has three EMS staff who can all perform cardiopulmonary resuscitation according to the Japanese guidelines; which, until September 2006, were based on the International Liaison Committee on Resuscitation and the American Heart Association 2000 Guidelines [16]. Since October 2006, resuscitation has been based on the respective 2005 Guidelines [14,17]. In Japan, EMS personnel can give only epinephrine and cannot give amiodarone.

### Automated external defibrillator

Each ambulance is equipped with one automated external defibrillator (AED). Either a monophasic or a biphasic defibrillator is applied to OHCA patients, according to the type of defibrillator on the EMS ambulance. The monophasic defibrillator models used in the present study were Heart Start 3000/3000QR (Laerdal Medical, Stavanger, Norway), LIFEPAK 12A (Medtronic), TEC 2202/2203/2212/2213, and AED-9100/9110 (Nihon Kohden). The monophasic defibrillators delivered either monophasic damped sine defibrillation waveforms or monophasic truncated exponential waveforms, according to the type of defibrillator on each EMS ambulance.

Biphasic defibrillator models were Heart Start MRxE/MRx, Heart Start FRx, Heart Start FR2/FR2+, Heart Start 4000, Heart Start HS1, Heart Start XL (Philips Medical Systems, Seattle, WA, USA), AED-9200/9210, AED-9231/9211/9201, AED-1200, AED-2100, TEC-2312/2313, TEC-2503/2513 (Nihon Kohden, Tokyo, Japan), or LIFEPAK 500B, LIFEPAK 1000, LIFEPAK 12B, and LIFEPAK CR-Plus (Medtronic, Minneapolis, MN, USA). All biphasic defibrillators in this study delivered the biphasic truncated exponential waveform.

EMS personnel used the both monophasic and biphasic defibrillators in AED mode, not manual mode. The defibrillation energy dose was set at the level for adults. The recommended adult dose of the monophasic defibrillator was initially 200 J, and thereafter, 360 J. The biphasic defibrillator energy dose was set at the level recommended by the manufacturer. For children aged 1 to 8 years, defibrillation was performed by using self-adhesive pad electrodes with pediatric attenuator systems. For children aged 9 to 17 years, a self-adhesive pad without pediatric attenuator systems was used in the same way as in adults.

### Data collection and quality control

All OHCA patient information was input by using an online entry form by EMS personnel, which basically conformed to the Utstein form, with some additions. The data were anonymized at the local fire departments and then transferred to the Fire and Disaster Management Agency (FDMA) and stored [18,19]. The data were checked, and if any were missing, the FDMA informed the corresponding regional fire department, and the data were corrected [18].

The main database items included patient age, sex, bystander-witness status, receipt of bystander CPR, receipt of defibrillation by the EMS, type of defibrillator, and the etiology of the cardiac arrest (cardiac or noncardiac origin). Outcome data included return of spontaneous circulation before arrival at the hospital, survival at 1 month, and neurologic status 1 month after the OHCA [20,21].

The etiology of cardiac arrest was determined by the physician in charge based on physical, laboratory, or radiologic findings, together with scene information obtained from EMS crew [22]. It was presumed to be cardiac in origin unless unequivocal evidence suggested respiratory diseases, cerebrovascular diseases, external causes (trauma, hanging, drowning, drug overdose, asphyxia) or any other noncardiac cause. One-month survival and neurologic- status data were collected by EMS personnel from the hospitals that received the patients, in cooperation with the physicians in charge of the patients through a follow-up interview at 1 month after hospital admission [14,20,23].

### Study targets and end points

In this study, we focused on pediatric patients who had confirmed shockable rhythms (ventricular fibrillation or pulseless ventricular tachycardia) and received defibrillation shock by EMS personnel. The primary outcome measure was survival at 1 month with minimal neurologic impairment, which was defined as Glasgow-Pittsburgh Cerebral Performance Category 1 (good cerebral performance) or 2 (moderate cerebral disability) [24,25]. The secondary outcome measures were survival at 1 month and the return of spontaneous circulation (ROSC) before hospital arrival.

### Statistical analysis

We compared the outcomes of pediatric OHCA patients receiving defibrillation shock with a monophasic waveform defibrillator with those receiving shock with a biphasic waveform defibrillator. Age was dichotomized into children aged 1 through 11 years (children) and 12 through 17 years (adolescents). The time from emergency call to CPR by EMS was divided into the following three categories: early response (0 to 6 minutes), moderate response (7 to 12 minutes), and late response (13 to 18 minutes). Based on the CPR type, the subjects were categorized into 2000 Guideline-based subgroup and 2005 Guideline-based subgroup. Patient characteristics were evaluated by using unpaired Student *t *tests for numeric variables and χ^2 ^tests for categoric variables. Outcomes by type of defibrillator were compared by using χ^2 ^tests.

To identify the association between defibrillator type (monophasic or biphasic) and outcomes, we performed multivariate logistic regression analyses with adjustment for age, sex, bystander-witness status, the type of bystander CPR (no bystander CPR, compression-only CPR or conventional CPR), time from emergency call to CPR by EMS (early, moderate, or late response), cause of arrest (cardiac or noncardiac), the type of guideline-based CPR performed (2000 Guideline based or 2005 Guideline based), and calendar year. We assumed that our data were structured hierarchically into two levels of patients and communities. We accounted for clustering of patients within communities by using a generalized estimation equation (GEE). This method is commonly used instead of a traditional regression analysis because patients in the same community may be correlated, thus violating independence assumptions made by traditional regression procedures [26]. All statistical analyses were conducted by using PASW ver. 17.0J (SPSS, Inc., Chicago, IL, USA). All tests were two-tailed, and a *P *value < 0.05 was regarded as significant.

## Results

The total number of OHCA patients (aged 1 through 17 years) was 5,628 during the study period. Patients who had no attempted resuscitation by EMS (*n *= 283), patients not shocked by EMS (*n *= 4848), patients lacking information on whether defibrillation shock was done or not (*n *= 43; < 0.8%), and patients who underwent defibrillation by a public-access AED by bystanders (*n *= 24; < 0.4%) were excluded. Consequently, the number of OHCA patients who were shocked for defibrillation by EMS was 430. These 430 patients were eligible for this study. Of the eligible patients, 127 (30%) received shocks with a monophasic defibrillator, and 303 (70%) received shocks with a biphasic defibrillator. Table 1 shows the demographic characteristics of the included pediatric patients. The proportion of patients receiving biphasic waveform defibrillation increased yearly.

**Table 1:** Patient characteristics of the study participants

	Total		Monophasic		Biphasic		*P*
Number of cases	(*n *= 430)		(*n *= 127)		(*n *= 303)		
Year							< 0.001
2005, *n *(%)	89	(20.7)	48	(37.8)	41	(13.5)	
2006, *n *(%)	74	(17.2)	30	(23.6)	44	(14.5)	
2007, *n *(%)	90	(20.9)	26	(20.5)	64	(21.1)	
2008, *n *(%)	92	(21.4)	15	(11.8)	77	(25.4)	
2009, *n *(%)	85	(19.8)	8	(6.3)	77	(25.4)	
Age, mean (SD)	12.8	(4.2)	13.3	(3.7)	12.6	(4.4)	0.08
Children (1 to 11 years old), *n *(%)	119	(27.7)	35	(27.6)	84	(27.7)	0.97
Adolescents (12 to 17 years old), *n *(%)	311	(72.3)	92	(72.4)	219	(72.3)	
Boys, *n *(%)	293	(68.1)	84	(66.1)	209	(69.0)	0.56
Witnessed by laypersons, *n *(%)	287	(66.7)	87	(68.5)	200	(66.0)	0.62
Type of bystander-initiated CPR^a^							0.05
No-CPR, *n *(%)	193	(45.2)	63	(50.0)	130	(43.2)	
Compression-only CPR, *n *(%)	97	(22.7)	19	(15.1)	78	(25.9)	
Conventional CPR, *n *(%)	137	(32.1)	44	(34.9)	93	(30.9)	
Call to CPR by EMS, minutes, mean (SD)^b^	8.9	(5.3)	8.8	(5.3)	9.0	(5.4)	0.34
0 to 6 minutes, *n *(%)	137	(32.0)	46	(36.5)	91	(30.1)	
7 to 12 minutes, *n *(%)	236	(55.1)	67	(53.2)	169	(56.0)	
13 to 18 min, *n *(%)	55	(12.8)	13	(10.4)	42	(13.9)	
CPR by EMS to hospital arrival, minutes, mean (SD)	21.3	(10.5)	21.6	(10.6)	21.1	(10.4)	0.66
Type of origin							0.72
Cardiac, *n *(%)	272	(63.3)	82	(64.6)	190	(62.7)	
Noncardiac, *n *(%)	158	(36.7)	45	(35.4)	113	(37.3)	
External causes, *n *(%)	92	(21.4)	24	(18.9)	68	(22.4)	
Respiratory diseases, *n *(%)	10	(2.3)	4	(3.1)	6	(2.0)	
Cerebrovascular diseases, *n *(%)	7	(1.6)	2	(1.6)	5	(1.7)	
Others, *n *(%)	49	(11.4)	15	(11.8)	34	(11.2)	
CPR guidelines							< 0.001
2000 Guideline based, *n *(%)	146	(34.0)	74	(58.3)	72	(23.8)	
2005 Guideline based, *n *(%)	284	(66.0)	53	(41.7)	231	(76.2)	
Number of shocks administered to patients who had ROSC before hospital arrival							
Median (25% to 75%)	1	(1-2)	1	(1-2)	1	(1-2)	0.76

CPR, cardiopulmonary resuscitation. ^a^Three (0.7%) patients with missing data were excluded. Percentages were calculated based on the total number of events, excluding those missing data. ^b^Two (0.5%) patients with missing data were excluded. Percentages were calculated based on the total number of events, excluding those missing data. AHA, American Heart Association; CPR, cardiopulmonary resuscitation; SD, standard deviation; EMS, emergency medical service.

Table 2 shows outcomes by type of defibrillator of eligible patients and of subgroups. Neurologic status was not documented for one patient in the monophasic waveform group. Chi-square tests showed no significant differences in any outcome measures between monophasic and biphasic groups in all patients and all subgroups.

**Table 2:** Outcomes of all patients and subgroups.

	ROSC before hospital arrival			Survival at 1 month			Survival at 1 month with minimal neurologic impairment		
	
	*n*	(%)	*P*	*n*	(%)	*P*	*n*	(%)	*P*
All patients (*n *= 430)									
Monophasic (*n *= 127)	31	24.4%	0.52	41	32.3%	0.50	22	17.5%	0.12
Biphasic (*n *= 303)	83	27.4%		108	35.6%		74	24.4%	
									
Age category									
Children (1 to 11 years)									
Monophasic (*n *= 35)	6	17.1%	0.10	9	25.7%	0.51	3	8.6%	0.72
Biphasic (*n *= 84)	6	7.1%		17	20.2%		9	10.7%	
Adolescents (12 to 17 years)									
Monophasic (*n *= 92)	25	27.2%	0.17	32	34.8%	0.27	19	20.9%	0.11
Biphasic (*n *= 219)	77	35.2%		91	41.6%		65	29.7%	
									
Time from emergency call to CPR by EMS									
Early response (0 to 6 minutes)									
Monophasic (*n *= 46)	13	28.3%	0.35	19	41.3%	0.65	11	24.4%	0.37
Biphasic (*n *= 91)	33	36.3%		34	37.4%		29	31.9%	
Moderate response (7 to 12 minutes)									
Monophasic (*n *= 67)	17	25.4%	0.93	21	31.3%	0.20	10	14.9%	0.12
Biphasic (*n *= 169)	42	24.9%		68	40.2%		41	24.3%	
Late response (13 to 18 minutes)									
Monophasic (*n *= 13)	1	7.7%	0.33	1	7.7%	0.53	1	7.7%	0.84
Biphasic (*n *= 42)	8	19.0%		6	14.3%		4	9.5%	
									
Origin of cardiac arrest									
Cardiac									
Monophasic (*n *= 82)	25	30.5%	0.61	32	39.0%	0.53	19	23.2%	0.059
Biphasic (*n *= 190)	64	33.7%		82	43.2%		66	34.7%	
Noncardiac									
Monophasic (*n *= 45)	6	13.3%	0.59	9	20.0%	0.68	3	6.8%	0.95
Biphasic (*n *= 113)	19	16.8%		26	23.0%		8	7.1%	
									
CPR Guidelines									
2000 Guideline based									
Monophasic (*n *= 74)	17	23.0%	0.12	24	32.4%	0.64	12	16.4%	0.50
Biphasic (*n *= 72)	25	34.7%		26	36.1%		15	20.8%	
2005 Guideline based									
Monophasic (*n *= 53)	14	26.4%	0.84	17	32.1%	0.64	10	18.9%	0.31
Biphasic (*n *= 231)	58	25.1%		82	35.5%		59	25.5%	

CPR, cardiopulmonary resuscitation; ROSC, return of spontaneous circulation.

Table 3 shows the results of logistic GEE regression analyses. No significant differences were found between the monophasic and biphasic groups in any outcome measures of eligible patients, including ROSC before hospital arrival (odds ratio (OR), 1.46; 95% confidence interval (CI), 0.8 to 2.63; *P *= 0.20), survival at 1 month (OR, 1.38; 95% CI, 0.87 to 2.18; *P *= 0.0.17), and survival at 1 month with minimal neurologic impairment (OR, 1.57; 95% CI, 0.87 to 2.83; *P *= 0.14).

**Table 3:** Odds ratios of outcomes for biphasic vs. monophasic defibrillators in the logistic regression models

	ROSC before hospital arrival					Survival at 1 month					Survival at 1 month with minimal neurologic impairment				
	
	OR	(95% CI)			*P*	OR	(95% CI)			*P*	OR	(95% CI)			*P*
Age (1-year increase)	1.13	1.06	to	1.21	0.009	1.05	0.98	to	1.11	0.17	1.11	1.03 to		1.20	0.01
Sex															
Boys	Ref.					Ref.					Ref.				
Girls	1.19	0.71	to	1.98	0.51	1.58	1.05	to	2.36	0.03	1.50	0.89	to	2.51	0.13
Witness status															
Witness	Ref.					Ref.					Ref.				
No witness	0.75	0.48 to	to	1.17	0.20	0.55	0.34 to		0.89	0.02	0.40	0.23	to	0.71	0.002
Type of bystander-initiated CPR															
No CPR	Ref.					Ref.					Ref.				
Compression-only CPR	0.81	0.50	to	1.31	0.40	0.85	0.50	to	1.45	0.56	1.32	0.72	to	2.41	0.37
Conventional CPR	1.34	0.87	to	2.06	0.18	1.38	0.91	to	2.08	0.13	1.54	0.87	to	2.73	0.14
Call to CPR by EMS															
0 to 6 minutes	Ref.					Ref.					Ref.				
7 to 12 minutes	0.68	0.44	to	1.06	0.09	1.00	0.65	to	1.53	0.99	0.67	0.36	to	1.27	0.22
13 to 18 minutes	0.46	0.21	to	1.01	0.05	0.26	0.12	to	0.58	0.001 >	0.35	0.12	to	0.97	0.04
Cause of arrest															
Cardiac origin	Ref.					Ref.					Ref.				
Noncardiac origin	0.48	0.28	to	0.83	0.01	0.51	0.31	to	0.85	0.01	0.23	0.11	to	0.49	0.001 >
Type of guideline-based CPR performed															
2000 Guideline based	Ref.					Ref.					Ref.				
2005 Guideline based	0.58	0.17	to	1.95	0.37	1.09	0.41	to	2.89	0.86	1.24	0.34	to	4.47	0.74
Year															
2005	Ref.					Ref.					Ref.				
2006	1.24	0.71	to	2.16	0.45	1.09	0.59	to	2.02	0.78	0.90	0.38	to	2.12	0.81
2007	1.65	0.36	to	7.45	0.52	0.84	0.30	to	2.34	0.74	1.13	0.27	to	4.75	0.86
2008	1.63	0.42	to	6.31	0.48	1.08	0.36	to	3.22	0.89	1.42	0.34	to	5.85	0.63
2009	1.98	0.55	to	7.12	0.29	0.92	0.29	to	2.86	0.88	1.32	0.33	to	5.22	0.70
Defibrillator															
Monophasic	Ref.					Ref.					Ref.				
Biphasic	1.46	0.81	to	2.63	0.20	1.38	0.87	to	2.18	0.17	1.57	0.87	to	2.83	0.14

All models included age, sex, whether the collapse was witnessed by a bystander, the type of bystander CPR performed (no bystander CPR, compression-only CPR, or conventional CPR), time from emergency call to CPR by the EMS (early response, moderate response, or late response), cause of arrest, the type of guideline-based CPR performed (2000 AHA Guideline based or 2005 AHA Guideline based), calendar year and the type of defibrillator (monophasic or biphasic). Regression analyses did not include patients with missing data. OR, odds ratio; CI, confidence interval.

## Discussion

Our study showed no significant differences in outcomes between the pediatric OHCA patients who were shocked with a biphasic defibrillator and those who were shocked with a monophasic defibrillator. So far, effective waveform types for defibrillation of pediatric VF have not been well determined. Only studies based on animal models report the potential effectiveness of certain types [27-29]. In an animal model of pediatric defibrillation with "infant" and "child" piglets, biphasic were more effective than monophasic waveforms [29]. Regarding human studies, previous studies with adult patients reported that biphasic waveform shock was superior to monophasic waveform shock in terms of safety under controlled laboratory and in-hospital conditions [6,8,9]. Regarding long-term outcomes, no significant difference was detected between the two types of waveform for defibrillation in any observational studies or in four randomized trials for adults [30-34]. In pediatric patients, no studies compared biphasic and monophasic waveforms, even in controlled laboratory or in-hospital settings. To the best of our knowledge, the present study is the first to verify the association of the outcomes and defibrillation with different defibrillator waveforms in pediatric OHCA.

Owing to the results of adult studies comparing the two defibrillators in laboratory and in-hospital settings, or the greater portability of biphasic defibrillators, most monophasic defibrillators were replaced with biphasic defibrillators in ambulances in Japan. This tendency is common worldwide. Biphasic defibrillators may reduce the physical burden on EMS personnel because of their greater portability. With respect to effectiveness, however, the present study showed no significant advantage of biphasic over monophasic defibrillators on meaningful clinical outcomes for pediatric patients.

### Study limitations

Several limitations of this study should be considered. First, because of the relatively small sample size (*n *= 430), the present study may have been too underpowered to detect true differences in outcomes between the groups. For example, to detect the true difference in 1-month survival with minimal neurologic impairment (17.5% versus 24.4%), the necessary sample size was estimated to be 1,321 on the basis of a two-sided α value of 0.05 and a β error of 0.20 [35].

Second, the inherent bias in an observational study is a potential limitation. For example, the monophasic EMS users were possibly less well supported by public funds, and received a lower frequency of training and retraining. If true, this could have influenced the results.

Third, data on whether VF was terminated by defibrillation were not collected, so our study could not directly compare the probability of terminating VF after defibrillation. However, 1-month survival with minimal neurologic impairment is considered to be a better outcome measure than the probability of terminating VF.

Fourth, the neurologic outcome in the database was not defined by pediatric Cerebral Performance Category [36].

However, despite these limitations, we believe that our study is valid, given the use of uniform data collection and consistent definitions based on the Utstein guidelines [24,25] and the relatively large sample size as an observational study of pediatric OHCA in a nationwide population-based setting.

## Conclusions

Our nationwide population-based observational study did not confirm an advantage of biphasic defibrillator over a monophasic defibrillator for 1-month survival with minimal neurologic impairment of pediatric OHCA patients.

## Key messages

• No significant differences in neurologic outcomes was found between the pediatric OHCA patients who were shocked with a biphasic defibrillator and those who were shocked with a monophasic defibrillator.

• Biphasic defibrillators may reduce the physical burden on EMS personnel because of their greater portability. With respect to effectiveness, however, the present study showed no significant advantage of biphasic over monophasic defibrillators on meaningful clinical outcomes for pediatric patients.

## Abbreviations

AED: automated external defibrillator; CPC: cerebral performance category; CPR: cardiopulmonary resuscitation; EMS: emergency medical service; FDMA: Fire and Disaster Management Agency of Japan; GEE: generalized estimation equation; OHCA: out-of-hospital cardiac arrest; OR: odds ratio; ROSC: return of spontaneous circulation; VF: ventricular fibrillation.

## Competing interests

The authors declare that they have no competing interests.

## Authors' contributions

ST and TI designed the study. TO and ST conducted data cleaning. HY, HH, and TH provided statistical advice on the study design and analyzed the data. ST drafted the manuscript, and ST, HY, TH, HY, TO, MA, and SK contributed substantially to its revision. ST takes responsibility for the article as a whole. All authors approved the manuscript before submission.

## References

[B1] Hickey RW Cohen DM Strausbaugh S Dietrich AM Pediatric patients requiring CPR in the prehospital setting Ann Emerg Med 1995 16 495 501 10.1016/S0196-0644(95)70265-2 7710155

[B2] Appleton GO Cummins RO Larson MP Graves JR CPR and the single rescuer: at what age should you "call first" rather than "call fast"? Ann Emerg Med 1995 16 492 494 10.1016/S0196-0644(95)70264-4 7710154

[B3] Ronco R King W Donley DK Tilden SJ Outcome and cost at a children's hospital following resuscitation for out-of-hospital cardiopulmonary arrest Arch Pediatr Adolesc Med 1995 16 210 214 10.1001/archpedi.1995.02170140092017 7849887

[B4] Losek JD Hennes H Glaeser P Hendley G Nelson DB Prehospital care of the pulseless, nonbreathing pediatric patient Am J Emerg Med 1987 16 370 374 10.1016/0735-6757(87)90383-4 3620034

[B5] Safranek DJ Eisenberg MS Larsen MP The epidemiology of cardiac arrest in young adults Ann Emerg Med 1992 16 1102 1106 10.1016/S0196-0644(05)80651-1 1514721

[B6] Osswald S Trouton TG O'Nunain SS Holden HB Ruskin JN Garan H Relation between shock-related myocardial injury and defibrillation efficacy of monophasic and biphasic shocks in a canine model Circulation 1994 16 2501 2509 10.1161/01.CIR.90.5.2501 7955208

[B7] Gliner BE Lyster TE Dillion SM Bardy GH Transthoracic defibrillation of swine with monophasic and biphasic waveforms Circulation 1995 16 1634 1643 10.1161/01.CIR.92.6.1634 7664451

[B8] Bardy GH Gliner BE Kudenchuk PJ Poole JE Dolack GL Jones GK Anderson J Troutman C Johnson G Truncated biphasic pulses for transthoracic defibrillation Circulation 1995 16 1768 1774 10.1161/01.CIR.91.6.1768 7882486

[B9] Bardy GH Marchlinski FE Sharma AD Worley SJ Luceri RM Yee R Halperin BD Fellows CL Ahern TS Chilson DA Packer DL Wilber DJ Mattioni TA Reddy R Kronmal RA Lazzara R Multicenter comparison of truncated biphasic shocks and standard damped sine wave monophasic shocks for transthoracic ventricular defibrillation: Transthoracic Investigators Circulation 1996 16 2507 2514 10.1161/01.CIR.94.10.2507 8921795

[B10] White RD Early out-of-hospital experience with an impedance-compensating low-energy biphasic waveform automatic external defibrillator J Interv Card Electrophysiol 1997 16 203 208 discussion 209-210 10.1023/A:1009712721915 9869972

[B11] Poole JE White RD Kanz KG Hengstenberg F Jarrard GT Robinson JC Santana V McKenas DK Rich N Rosas S Merritt S Magnotto L Gallagher JV Gliner BE Jorgenson DB Morgan CB Dillon SM Kronmal RA Bardy GH Low-energy impedance-compensating biphasic waveforms terminate ventricular fibrillation at high rates in victims of out-of-hospital cardiac arrest: LIFE Investigators J Cardiovasc Electrophysiol 1997 16 1373 1385 10.1111/j.1540-8167.1997.tb01034.x 9436775

[B12] Gliner BE Jorgenson DB Poole JE White RD Kanz KG Lyster TD Leyde KW Powers DJ Morgan CB Kronmal RA Bardy GH Treatment of out-of-hospital cardiac arrest with a low-energy impedance-compensating biphasic waveform automatic external defibrillator: The LIFE Investigators Biomed Instrum Technol 1998 16 631 644 9883348

[B13] Link MS Atkins DL Passman RS Halperin HR Samson RA White RD Cudnik MT Berg MD Kudenchuk PJ Kerber RE Part 6: Electrical therapies: automated external defibrillators, defibrillation, cardioversion, and pacing: 2010 American Heart Association Guidelines for Cardiopulmonary Resuscitation and Emergency Cardiovascular Care Circulation 2010 16 S706 S719 10.1161/CIRCULATIONAHA.110.970954 20956222

[B14] Kitamura T Iwami T Kawamura T Nagao K Tanaka H Hiraide A Nationwide public-access defibrillation in Japan N Engl J Med 2010 16 994 1004 10.1056/NEJMoa0906644 20237345

[B15] Tanigawa K Tanaka K Emergency medical service systems in Japan: past, present, and future Resuscitation 2006 16 365 370 10.1016/j.resuscitation.2006.04.001 16740355

[B16] Guidelines 2000 for Cardiopulmonary Resuscitation and Emergency Cardiovascular Care. Part 6: advanced cardiovascular life support: section 8: postresuscitation care: The American Heart Association in collaboration with the International Liaison Committee on Resuscitation Circulation 2000 16 I166 I171 10966672

[B17] 2005 American Heart Association Guidelines for Cardiopulmonary Resuscitation and Emergency Cardiovascular Care Circulation 2005 16 IV1 203 16314375 10.1161/CIRCULATIONAHA.105.166550

[B18] Ogawa T Akahane M Koike S Tanabe S Mizoguchi T Imamura T Outcomes of chest compression only CPR versus conventional CPR conducted by lay people in patients with out of hospital cardiopulmonary arrest witnessed by bystanders: nationwide population based observational study BMJ 2010 16 c7106 10.1136/bmj.c7106 21273279

[B19] Yasunaga H Horiguchi H Tanabe S Akahane M Ogawa T Koike S Imamura T Collaborative effects of bystander-initiated cardiopulmonary resuscitation and prehospital advanced cardiac life support by physicians on survival of out-of-hospital cardiac arrest: a nationwide population-based observational study Crit Care 2010 16 R199 10.1186/cc9319 21050434 PMC3219985

[B20] Akahane M Ogawa T Tanabe S Koike S Horiguchi H Yasunaga H Imamura T Impact of telephone dispatcher assistance on the outcomes of pediatric out-of-hospital cardiac arrest Crit Care Med 2012 16 1410 1416 10.1097/CCM.0b013e31823e99ae 22430245

[B21] Koike S Ogawa T Tanabe S Matsumoto S Akahane M Yasunaga H Horiguchi H Imamura T Collapse-to-emergency medical service cardiopulmonary resuscitation interval and outcomes of out-of-hospital cardiopulmonary arrest: a nationwide observational study Crit Care 2011 16 R120 10.1186/cc10219 21545735 PMC3218973

[B22] Tanabe S Ogawa T Akahane M Koike S Horiguchi H Yasunaga H Mizoguchi T Hatanaka T Yokota H Imamura T Comparison of neurological outcome between tracheal intubation and supraglottic airway device insertion of out-of-hospital cardiac arrest patients: a nationwide, population-based, observational study J Emerg Med 2012 in press 10.1016/j.jemermed.2012.02.026 22541878

[B23] Hagihara A Hasegawa M Abe T Nagata T Wakata Y Miyazaki S Prehospital epinephrine use and survival among patients with out-of-hospital cardiac arrest JAMA 2012 16 1161 1168 10.1001/jama.2012.294 22436956

[B24] Cummins RO Chamberlain DA Abramson NS Allen M Baskett PJ Becker L Bossaert L Delooz HH Dick WF Eisenberg MS Recommended guidelines for uniform reporting of data from out-of-hospital cardiac arrest: the Utstein Style. A statement for health professionals from a task force of the American Heart Association, the European Resuscitation Council, the Heart and Stroke Foundation of Canada, and the Australian Resuscitation Council Circulation 1991 16 960 975 10.1161/01.CIR.84.2.960 1860248

[B25] Jacobs I Nadkarni V Bahr J Berg RA Billi JE Bossaert L Cassan P Coovadia A D'Este K Finn J Halperin H Handley A Herlitz J Hickey R Idris A Kloeck W Larkin GL Mancini ME Mason P Mears G Monsieurs K Montgomery W Morley P Nichol G Nolan J Okada K Perlman J Shuster M Steen PA Sterz F Cardiac arrest and cardiopulmonary resuscitation outcome reports: update and simplification of the Utstein templates for resuscitation registries: a statement for healthcare professionals from a task force of the International Liaison Committee on Resuscitation (American Heart Association, European Resuscitation Council, Australian Resuscitation Council, New Zealand Resuscitation Council, Heart and Stroke Foundation of Canada, InterAmerican Heart Foundation, Resuscitation Councils of Southern Africa) Circulation 2004 16 3385 3397 10.1161/01.CIR.0000147236.85306.15 15557386

[B26] Hubbard AE Ahern J Fleischer NL Van der Laan M Lippman SA Jewell N Bruckner T Satariano WA To GEE or not to GEE: comparing population average and mixed models for estimating the associations between neighborhood risk factors and health Epidemiology 2010 16 467 474 10.1097/EDE.0b013e3181caeb90 20220526

[B27] Berg RA Chapman FW Berg MD Hilwig RW Banville I Walker RG Nova RC Sherrill D Kern KB Attenuated adult biphasic shocks compared with weight-based monophasic shocks in a swine model of prolonged pediatric ventricular fibrillation Resuscitation 2004 16 189 197 10.1016/j.resuscitation.2003.12.021 15135196

[B28] Tang W Weil MH Jorgenson D Klouche K Morgan C Yu T Sun S Snyder D Fixed-energy biphasic waveform defibrillation in a pediatric model of cardiac arrest and resuscitation Crit Care Med 2002 16 2736 2741 10.1097/00003246-200212000-00019 12483066

[B29] Clark CB Zhang Y Davies LR Karlsson G Kerber RE Pediatric transthoracic defibrillation: biphasic versus monophasic waveforms in an experimental model Resuscitation 2001 16 159 163 10.1016/S0300-9572(01)00398-7 11718971

[B30] Tanabe S Yasunaga H Ogawa T Koike S Akahane M Horiguchi H Hatanaka T Yokota H Imamura T Comparison of outcomes after use of biphasic or monophasic defibrillators among out-of-hospital cardiac arrest patients: a nationwide population-based observational study Circ Cardiovasc Qual Outcomes 2012 16 689 696 22967787 10.1161/CIRCOUTCOMES.112.965319

[B31] Schneider T Martens PR Paschen H Kuisma M Wolcke B Gliner BE Russell JK Weaver WD Bossaert L Chamberlain D Multicenter, randomized, controlled trial of 150-J biphasic shocks compared with 200- to 360-J monophasic shocks in the resuscitation of out-of-hospital cardiac arrest victims: Optimized Response to Cardiac Arrest (ORCA) Investigators Circulation 2000 16 1780 1787 10.1161/01.CIR.102.15.1780 11023932

[B32] Morrison LJ Dorian P Long J Vermeulen M Schwartz B Sawadsky B Frank J Cameron B Burgess R Shield J Bagley P Mausz V Brewer JE Lerman BB Out-of-hospital cardiac arrest rectilinear biphasic to monophasic damped sine defibrillation waveforms with advanced life support intervention trial (ORBIT) Resuscitation 2005 16 149 157 10.1016/j.resuscitation.2004.11.031 15992986

[B33] van Alem AP Chapman FW Lank P Hart AA Koster RW A prospective, randomised and blinded comparison of first shock success of monophasic and biphasic waveforms in out-of-hospital cardiac arrest Resuscitation 2003 16 17 24 10.1016/S0300-9572(03)00106-0 12867305

[B34] Kudenchuk PJ Cobb LA Copass MK Olsufka M Maynard C Nichol G Transthoracic incremental monophasic versus biphasic defibrillation by emergency responders (TIMBER): a randomized comparison of monophasic with biphasic waveform ascending energy defibrillation for the resuscitation of out-of-hospital cardiac arrest due to ventricular fibrillation Circulation 2006 16 2010 2018 10.1161/CIRCULATIONAHA.106.636506 17060379

[B35] Hulley SB, Cummings SR, Browner WS, Grady DG, Newman TB Estimating sample size and power Designing Clinical Research 2006 3 Philadelphia: Lippincott Williams & Wilkins Chapter 6

[B36] Fiser DH Assessing the outcome of pediatric intensive care J Pediatr 1992 16 68 74 10.1016/S0022-3476(05)82544-2 1625096

